# Poisonous Moulds

**Published:** 1869-03

**Authors:** 


### POISONOUS MOULDS.

Moulded bread, meat, cheese, or any other eatable is an actual poison, whether inhaled or eaten. One kind of mould causes the fatal ship-fever. The mould in damp cellars causes various grades of typhoid fever, diarrhœa, dysentery, etc. Recent chemical researches and microscopic observation seem to show that miasm is nothing more or less than a mould, and that this mould is, in reality, a cloud of living things, each too small to be seen by the naked eye, and are drawn into the lungs, swallowed with the saliva, incorporated with the food eaten, and by being absorbed into the blood are sufficient to cause all grades of deadly fevers; elevated or dry localities are wholly exempt.

